# Psychometric properties of the Parma Scale for the treatment evaluation of prisoners with mental disorder: a new instrument for routine outcome monitoring in different forensic psychiatric settings

**DOI:** 10.1192/j.eurpsy.2023.924

**Published:** 2023-07-19

**Authors:** L. Pelizza, G. Paulillo, P. Pellegrini

**Affiliations:** Department of Mental Health, AUSL di Parma, Parma, Italy

## Abstract

**Introduction:**

The clinical relevance of Routine Outcome Monitoring (ROM) to formulate longitudinal evaluations of treatment appropriateness/efficacy and to assist decision making aimed at improving the quality of person-centered interventions has been poorly implemented in forensic psychiatry, also in Italy. Indeed, very few assessment instruments have been developed in this crucial field.

**Objectives:**

As reliable ROM instruments are lacking, the aim of the current investigation was to examine psychometric properties (i.e. reliability, concurrent validity and sensitivity to measure scores’ longitudinal changes) of the Parma Scale (Pr-Scale) (a new instrument fro the evaluation of offenders with mental disorder) in an Italian sample of forensic psychiatric patients.

**Methods:**

Participants were male adult offenders with mental disorder recruited within the Parma REMS (“Residence for the Execution of Security Measure”) or the Parma Penitentiary Institute (PPI). Exclusion criteria were known moderate/severe intellectual disability or any other medical condition inducing inability to express a valid consent for participating in the research. The Pr-Scale includes 20 items divided into 3 main domains: “Historical”, “Clinical” (observational) and “Treatment Plannining". To test psychometric properties of the Pr-Scale, we examined interrater reliability, short-term (1-week) test-retest reliability and internal consistency. As measure of concurrent validity, a correlation analysis of Pr-Scale item scores with corresponding HKT-R (the “Historisch, Klinische en Toekomstige – Revisie” instrument) item subscores was performed. Finally, we examined the Pr-Scale sensitivity to measure scores’ longitudinal changes over a 3-month treatment follow-up period.

**Results:**

60 male adult patients were recruited in this study. Our findings showed good to excellent interrater and test-retest reliability, concurrent validity and internal consistency for the Pr-Scale. Pr-Scale scores also display a moderate to large changeability over time (Intra-Class Correlation coefficient = 0.963, coefficient of stability = 0.997, Cronbach’s ɑ = 0.736). Statistically significant correlations of Pr-Scale item scores with the corresponding HKT-R scores were found. Across the 3-month follow-up period, we observed statistically significant sensitivity values in measuring longitudinal changes for the Pr-Scale total score and Pr-Scale domain and item subscores.

**Conclusions:**

Our results support the clinical use of the Pr-Scale in different forensic psychiatric settings (i.e. prison, REMS) as reliable ROM instrument.

**Disclosure of Interest:**

None Declared

